# Paclitaxel-Loaded Lipid-Coated Magnetic Nanoparticles for Dual Chemo-Magnetic Hyperthermia Therapy of Melanoma

**DOI:** 10.3390/pharmaceutics15030818

**Published:** 2023-03-02

**Authors:** Relton R. Oliveira, Emílio R. Cintra, Ailton A. Sousa-Junior, Larissa C. Moreira, Artur C. G. da Silva, Ana Luiza R. de Souza, Marize C. Valadares, Marcus S. Carrião, Andris F. Bakuzis, Eliana M. Lima

**Affiliations:** 1 FarmaTec—Laboratory of Pharmaceutical Technology, School of Pharmacy, Federal University of Goiás, Alameda Flamboyant, Qd. K, Ed. LIFE, Parque Tecnológico Samambaia, Goiânia 74690-631, Brazil; 2 ToxIn—Laboratory of Education and Research in In Vitro Toxicology, Federal University of Goiás, Alameda Flamboyant, Qd. K, Ed. LIFE, Parque Tecnológico Samambaia, Goiânia 74690-631, Brazil; 3 Physics Institute, Federal University of Goiás, Avenida Esperança, s/n, Campus Samambaia, Goiânia 74690-900, Brazil; 4 CNanoMed—Nanomedicine Integrated Research Center, Federal University of Goiás, Alameda Flamboyant, Qd. K, Ed. LIFE, Parque Tecnológico Samambaia, Goiânia 74690-631, Brazil

**Keywords:** paclitaxel, magnetosomes, triggered release, magnetic hyperthermia, B16F10 cells

## Abstract

Melanoma is the most aggressive and metastasis-prone form of skin cancer. Conventional therapies include chemotherapeutic agents, either as small molecules or carried by FDA-approved nanostructures. However, systemic toxicity and side effects still remain as major drawbacks. With the advancement of nanomedicine, new delivery strategies emerge at a regular pace, aiming to overcome these challenges. Stimulus-responsive drug delivery systems might considerably reduce systemic toxicity and side-effects by limiting drug release to the affected area. Herein, we report the development of paclitaxel-loaded lipid-coated manganese ferrite magnetic nanoparticles (PTX-LMNP) as magnetosomes synthetic analogs, envisaging the combined chemo-magnetic hyperthermia treatment of melanoma. PTX-LMNP physicochemical properties were verified, including their shape, size, crystallinity, FTIR spectrum, magnetization profile, and temperature profile under magnetic hyperthermia (MHT). Their diffusion in porcine ear skin (a model for human skin) was investigated after intradermal administration via fluorescence microscopy. Cumulative PTX release kinetics under different temperatures, either preceded or not by MHT, were assessed. Intrinsic cytotoxicity against B16F10 cells was determined via neutral red uptake assay after 48 h of incubation (long-term assay), as well as B16F10 cells viability after 1 h of incubation (short-term assay), followed by MHT. PTX-LMNP-mediated MHT triggers PTX release, allowing its thermal-modulated local delivery to diseased sites, within short timeframes. Moreover, half-maximal PTX inhibitory concentration (IC_50_) could be significantly reduced relatively to free PTX (142,500×) and Taxol^®^ (340×). Therefore, the dual chemo-MHT therapy mediated by intratumorally injected PTX-LMNP stands out as a promising alternative to efficiently deliver PTX to melanoma cells, consequently reducing systemic side effects commonly associated with conventional chemotherapies.

## 1. Introduction

Skin cancers are usually classified as either non-melanoma or melanoma. In global cancer statistics, non-melanoma skin cancers are often not reported since their incidence is extremely frequent worldwide relative to all the other cancer types. Additionally, since instances of non-melanoma skin cancers are frequently treated by primary care clinicians, the risks posed by such cancers tend to be underreported [1,2]. In contrast, melanoma is the 17th most common cancer worldwide [2]. It represents almost half of the cases among white populations. Of all skin cancers, melanoma is the most aggressive form, affecting deeper layers of the skin and having greater potential for metastasis [3].

Anti-melanoma therapies based on conventional chemotherapy are frequently ineffective. Therefore, alternatives to improve the prognostics have been explored. Some studies have demonstrated the efficacy of paclitaxel (PTX), a triterpene drug extracted from *Taxus brevifolia*, as a single agent or in combination with carboplatin to treat metastatic melanoma [4,5,6,7]. PTX inhibits cell growth by interacting with the microtubules through a non-covalent bond with tubulins, thus blocking mitosis [8,9].

Despite its potent antiproliferative effects, PTX presents major drawbacks, including systemic toxicity, due to its non-selective distribution to normal cells, and low water solubility. Hence, encapsulation of PTX into drug delivery systems, such as liposomes [10,11,12], magnetic nanoparticles [13,14,15], polymeric nanoparticles [16,17], solid lipid nanoparticles [18,19,20], as well as micelles [21,22], has been extensively investigated as an alternative to commercial formulations available in the market (Abraxane^®^ and Taxol^®^). Additionally, strategies comprising the combination of chemotherapeutic agents with other therapeutic modalities, such as photoinduced (e.g., photodynamic therapy) and heat-mediated therapies (e.g., photothermal therapy and magnetic hyperthermia) have been assessed, aiming to enhance treatment efficacy, while reducing chemotherapy side effects [23,24,25,26,27,28].

In particular, magnetic hyperthermia (MHT) emerges as a promising technique to treat cancers, based on its selective heat delivery to tumor cells, leading to cell death, either as a monotherapy, with the magnetothermal converting agents intratumorally injected so as to optimize the thermal dose, or in combination with chemotherapy or radiation therapy [27]. In MHT, an alternating magnetic field (AMF) is applied to the tumor region, where magnetic nanoparticles were previously injected [27,29]. Systemic administration is a major challenge, since interactions of nanoparticles with non-targeted tissues, particularly along the bloodstream, lead to nonspecific and inefficient delivery, consequently hindering the induction of thermal doses within therapeutic ranges at biologically safe regimes [30,31,32,33].

Excited by the AMF, the magnetic nanoparticles dissipate heat as a result of the interaction of their magnetic moments with the AMF, inducing a biological response. This response can range from a change in transport processes across the cell membranes to a modulation in the expression of specific molecules, eventually culminating with cell death by apoptosis or necrosis, depending on the thermal dose [27,29,34,35,36]. Clinical use of MHT, however, depends on a relatively homogeneous distribution of magnetic nanoparticles with improved SLP (specific loss power) within a significant volume of the tumor tissue [32]. Moreover, the range of field amplitudes and frequencies allowed for biologically safe conditions is limited, so as to prevent nonspecific heating (induced by eddy currents), as well as peripheral nerve stimulation and even cardiac muscle stimulation [32,33]. Thereby, MHT is currently being used in the clinic in combination with other therapies. Particularly, researchers have combined chemotherapy agents and magnetic nanoparticles aiming to enhance the pharmacological efficacy of the former against various types of cancer via dual chemo-MHT therapy [37,38,39,40,41].

Nevertheless, to fulfill these purposes, magnetic nanoparticles must exhibit specific properties, which are dependent on their size, shape, composition, collective organization, as well as on the frequency and amplitude of the AMF to which they are submitted [25,27,28,42]. An interesting approach, which has received increasing attention, is the use of magnetosomes in MHT [43,44,45,46,47]. Magnetosomes are magnetic nanoparticles (especially magnetite, Fe_3_O_4_) coated by a phospholipid bilayer, a product of magnetotactic bacteria [48]. Due to their biological nature, they are considered biocompatible and safe when used as a delivery framework [49]. However, the industrial-scale biosynthesis of magnetosomes would be a challenging task, justifying the search for synthetic analogs.

Herein, we report the development and characterization of thermoresponsive PTX-loaded lipid-coated magnetic nanoparticles (PTX-LMNP) that are conceptually synthetic analogs of magnetosomes. Their distribution in a skin model after intradermal injection was investigated. The influence of MHT both on their PTX release profiles and on their cytotoxicity against B16F10 melanoma cells were assessed. Our two initial hypotheses were: (1) PTX release from PTX-LMNP can be modulated by temperature (hence, by MHT) and (2) the combination of chemotherapy and MHT will result in an enhanced cytotoxicity against B16F10 melanoma cells.

## 2. Materials and Methods

### 2.1. Materials

Methylamine, manganese II chloride (MnCl_2_∙4H_2_O), iron III chloride (FeCl_3_∙6H_2_O), iron III nitrate (Fe(NO_3_)_3_∙9H_2_O), sodium oleate (SO), rhodamine, Dulbecco’s Modified Eagle Medium (DMEM), fetal bovine serum (FBS), trypsin-EDTA, Hoechst stain solution, and neutral red were purchased from Sigma Aldrich^®^ (St, Louis, MO, USA). Soy phosphatidylcholine (PC) was bought from Lipoid^®^ (Ludwigshafen, Germany). Paclitaxel (PTX) was acquired from LC Laboratories (United States). Penicillin/streptomycin was obtained from Gibco™ (Waltham, MA, USA). HPLC grade solvents were purchased from J.T. Baker (Radnor, PA, USA). B16F10 murine melanoma cells were provided by Banco de Células do Rio de Janeiro (BCRJ, Rio de Janeiro, Brazil). Ultrapure Milli-Q water was used throughout the experiments. All other reagents and solvents used were of analytical grade of superior.

### 2.2. Synthesis of Manganese Ferrite (MnFe_2_O_4_) Magnetic Nanoparticles (MNP)

Manganese ferrite (MnFe_2_O_4_) magnetic nanoparticles (MNP) were prepared by co-precipitation in methylamine, as previously reported [50]. Briefly, 90 mL of methylamine were first diluted in 400 mL of ultrapure water. Continuous magnetic stirring and heating were applied until boiling. Then, 50 mL of MnCl_2_ (0.5 M) and 50 mL of FeCl_3_ (1 M) aqueous solutions were poured in. Upon boiling, the mixture remained under stirring and heating for 30 min, during which a dark precipitate gradually formed. The ensemble was then left for cooling at room temperature for additional 30 min, at the end of which the precipitate containing MnFe_2_O_4_ MNP was magnetically separated and washed three times in water.

To protect the MNP surfaces from corrosion and oxidation, passivation with Fe(NO_3_)_3_ was performed, as previously described [50,51]. Briefly, 50 mL of HNO_3_ (0.5 M) and 50 mL of Fe(NO_3_)_3_ (0.5 M) aqueous solutions were poured on the MNP precipitate, followed by magnetic stirring and heating until boiling. Upon boiling, the mixture was left under stirring and heating for 30 min, at the end of which the ensemble was allowed to cool down. The passivated MNP were then magnetically separated and washed three times in acetone. Most of the MNP were stored in the fridge for later use, still in acetone. For characterization purposes, however, part of the synthesized MNP were further coated with citrate ions and resuspended in water, as previously reported [50].

### 2.3. Characterization of MnFe_2_O_4_ MNP

MnFe_2_O_4_ MNP aliquots were submitted to transmission electron microscopy (TEM, Jeol, JEM-2100, Thermo Scientific, Waltham, MA, USA), so that their shape and size distribution could be verified. Additionally, their crystallinity and size were assessed via X-ray diffraction (XRD, XRD-6000, Shimadzu, Kyoto, Japan). Mean diameters were then calculated via Scherrer’s equation:

(1) $$D=\frac{k\lambda }{\beta \cos\theta }$$

where k is the Scherrer’s constant (0.89), λ is the wavelength of the X-ray beam (0.154 nm), β is the full width at half-maximum (in rad) of the diffractogram peak under analysis, and θ is its corresponding Bragg’s angle [52].

Magnetization profiles of both powder and fluid samples were obtained by vibrating sample magnetometry (VSM, EV9 Magnetometer, ADE Magnetics). Specific saturation magnetizations of these samples were then used to determine the MNP colloid concentration, as previously reported [50]. The behavior of powder samples under magnetic hyperthermia (MHT) was observed for different alternating magnetic field (AMF) amplitudes (100–342 Oe) at 310 kHz (MagneTherm, NanoTherics^®^). Temperature measurements were performed using a fiber optic temperature sensor (Luxtron^®^).

Specific loss power (SLP) values were obtained (in W/g) as follows:

(2) $$\mathrm{SLP}=\frac{\rho c}{x}{\left(\frac{\mathrm{dT}}{\mathrm{dt}}\right)}_{t\to 0}$$

where ρ and c represent, respectively, the density (g/mL) and the specific heat capacity (Jg^−1^ °C^−1^) of the sample (both assumed to be approximately equal to those of the dispersant, for colloidal dispersions), whereas x denotes the concentration of magnetic material in the sample (g/mL), and ${\left(\mathrm{dT}/\mathrm{dt}\right)}_{t\to 0}$ is the rate at which temperature T (°C) changes right at the beginning of the experiment (in practice, the first 30 s of MHT).

### 2.4. Preparation of PTX-Loaded Lipid-Coated Magnetic Nanoparticles (PTX-LMNP)

PTX-loaded lipid-coated magnetic nanoparticles (PTX-LMNP) were prepared by lipid film hydration followed by probe sonication—an adaption of a previously reported method, originally designed to prepare magnetic solid lipid nanoparticles [15].

First, 0.5 mL of the previously stored suspension of MnFe_2_O_4_ MNP in acetone was added to 0.5 mL of chloroform containing 200 mg of phosphatidylcholine (PC) and 5 mg of paclitaxel (PTX). The mixture was then probe-sonicated (40 Hz, 5 min) to produce a homogeneous dispersion. The amount of MNP in this suspension was found to be 50 mg.

Then, the dispersion was transferred to a round bottom flask and the organic solvents were removed at room temperature and low pressure via rotary evaporation (IKA). After complete solvent evaporation, the MNP/PC/PTX film was left to dry under vacuum conditions overnight.

Next, the film was hydrated with 4 mL of an aqueous dispersion of sodium oleate (SO, 5 mg/mL) and probe-sonicated intermittently (40 Hz, 5 min) in an ice bath to form a homogeneous dispersion of PTX-LMNP. The resulting PTX-LMNP colloid was then filtered through a 0.22-μm syringe filter and centrifuged to remove traces of water-insoluble reagents.

For fluorescence microscopy experiments, about 1% (*w*/*w*) of rhodamine was incorporated to the PTX-LMNP, by adding 2 mg of this fluorophore to the MNP/PC/PTX mixture in acetone/chloroform whilst preparing the lipid film, as described here above.

For the preparation of freeze-dried (lyophilized) samples, microcrystalline trehalose was used as cryoprotectant, initially at 1:1 up to 1:6 (trehalose:PC, *w*/*w*). The freeze-dryer MicroModulyo (ThermoFisher) was used for the lyophilization procedures.

### 2.5. Characterization of PTX-LMNP

PTX-LMNP mean hydrodynamic sizes and polydispersity indexes were determined via dynamic light scattering (DLS, Zetasizer Nano S, Malvern, UK).

PTX quantitation was carried out by a high performance liquid chromatography equipment (HPLC Varian Pro Star) coupled to an automated sampler and to an UV detector, following a previously reported protocol [15]. The chromatographic runs were performed through a Zorbax Eclipse XDB-C18 column (particle size: 3.5 μm; length × internal diameter: 150 mm × 4.6 mm). The mobile phase consisted of acetonitrile, methanol, and water (50/10/40, *v*/*v*/*v*). The flow rate was 1 mL/min, and the column was kept at 25 °C during the runs. Detection was set at a wavelength of 227 nm. The injection volume was 20 μL. This analytical method was validated, showing selectivity, linearity within 0.5–50 μg/mL (r^2^ > 0.99), precision (relative standard deviation, RSD < 5%), and accuracy (>95%).

For PTX extraction, prior to HPLC quantitation, 100 μL of PTX-LMNP were poured into 1 mL of methanol, followed by sonication (10 min) and centrifugation (14,500 rpm, 10 min). The supernatant was filtered through a 0.45 μm and then submitted to HPLC. PTX entrapment efficiency (EE) and drug loading (DL) were determined as follows:

(3) $$\mathrm{EE}\;(\%)=100\times \frac{\mathrm{PTX}\;\mathrm{mass}\;\mathrm{entrapped}}{\mathrm{initial}\;(\mathrm{total})\;\mathrm{PTX}\;\mathrm{mass}}$$

(4) $$\mathrm{DL}\;(\%)=100\times \frac{\mathrm{PTX}\;\mathrm{mass}\;\mathrm{entrapped}}{\mathrm{total}\;\mathrm{mass}\;\mathrm{of}\;\mathrm{structural}\;\mathrm{lipids}\;(\mathrm{PC})}$$

To evaluate PTX-LMNP stability, lyophilized samples were stored at 4 °C for 30 days, whereas fluid samples were stored at 25 °C for 60 days. Then, mean hydrodynamic size, polydispersity index, and EE were determined. Lyophilized samples were reconstituted with ultrapure water.

To attest the success of PTX-LMNP assembly, Fourier Transform Infrared spectroscopy analyses (FTIR, Varian 640-IR) were carried out both for PTX-LMNP and each of its constituents (PTX, MNP, PC, and SO).

Magnetization profiles for both freeze-dried (lyophilized) and colloidally dispersed (fluid) samples were obtained by VSM.

The behavior of PTX-LMNP samples (1 mL at 3.5 mg/mL, in terms of MNP contents) under MHT was observed for different AMF amplitudes (100–305 Oe) at 310 kHz. Whenever needed, PC liposomes were used as non-magnetic controls.

### 2.6. PTX-LMNP Distribution in Porcine Ear Skin

To evaluate the distribution of PTX-LMNP in skin, 0.5 µL of PTX-LMNP labeled with rhodamine (about 1%) was injected intradermally. Porcine ear skin, an in vitro model for human skin [53], was placed on the top of a vertical diffusion cell. The receptor medium consisted of saline 0.9%, kept at 32 °C. After 2 h, an area of 50 mm^2^ of skin was harvested. The tissue was embedded in Tissue-Tek^®^ gel and frozen in N_2_ liquid. Next, 5-µm thick cryostat transversal sections were obtained using a Leica CM1850 cryomicrotome and mounted directly on microscope slides. The sections were stained with Hoechst (1 ng/mL) for 5 min. Then, samples were observed on a Leica DMI4000 B fluorescence microscope.

### 2.7. PTX-LMNP In Vitro Drug Release Profile

In vitro drug release profiles were obtained via diffusion through dialysis membrane. PTX quantitation was performed via HPLC. Briefly, 1 mL of PTX-LMNP (comprising approximately 1 mg of PTX and 3.5 mg of MNP) was placed in a dialysis bag (MWCO:14 kDa). Then, the bag was placed in a flask containing 20 mL of receptor medium, consisting of isopropanol:saline 0.9% (30:70, *v*/*v*). The flask was kept in a temperature-controlled orbital shaker incubator at 25 °C, 37 °C, or 43 °C for 96 h. Aliquots of 500 µL were withdrawn for PTX quantitation via HPLC at scheduled times—the withdrawn volume being replenished each time with fresh receptor medium.

Additionally, to assess the influence of MHT on PTX release, firstly, an AMF (310 kHz, 305 Oe) was applied to samples for about 40 min. After this single session of MHT, samples were then placed in dialysis bags, which were in turn placed in flasks containing the receptor medium and kept under orbital shaking at 25 °C for 96 h, as described here above. Similarly, aliquots were withdrawn for PTX quantitation at scheduled times with replenishment of the receptor medium after each withdrawal. No additional sessions of MHT were applied to the samples prior to PTX quantitation via HPLC.

All experiments were performed in quintuplicate. Cumulative PTX release profiles were expressed in terms of percentages of the total entrapped PTX. Data were then fit to Baker–Lonsdale, Peppas, Hixson–Crowell, Higuchi, and first-order models. The adjusted coefficient of determination (R^2^) was calculated for each case, and the best fits were used as predictive models of the corresponding drug release profiles.

### 2.8. PTX-LMNP Cytotoxicity against B16F10 Melanoma Cells

B16F10 cells (4 × 10^4^ cells/mL) were seeded on 96-well plates (100 μL/well) and incubated for 24 h at 37 °C under 5% CO_2_ atmosphere and controlled humidity. Next, cells were treated with liposomes, LMNP, or PTX-LMNP at concentrations ranging from 1:10 down to 1:10^8^ (*v*/*v*) relatively to the volume of culture medium (DMEM supplemented with 2% FBS), enabling the determination of the half-maximal inhibitory concentration (IC_50_) for each formulation. Cells were incubated for additional 48 h and then submitted to neutral red uptake assay [54]. Cells incubated only with culture medium were used as control samples and served as a reference for 100% of cell viability. Results were averaged over three independent experiments, each performed in sextuplicate.

The influence of MHT on PTX-LMNP cytotoxicity against B16F10 melanoma cells was assessed via trypan blue assay. First, cells were cultured in DMEM supplemented with 2% FBS and 1% penicillin/streptomycin. Culture flasks were incubated for 24 h at 37 °C under 5% CO_2_ atmosphere and controlled humidity. Next, cells were detached from the flask bottom using trypsin-EDTA (0.25–0.03%), resuspended in DMEM supplemented with 2% FBS, and divided into four aliquots (4 × 10^7^ cells/mL each). Aliquots labeled PTX-LMNP+ were treated with 10% PTX-LMNP (*v*/*v*), up to a final volume of 10 mL. About 1 h after incubation, which allowed sample homogenization and some PTX-LMNP internalization by the B16F10 cells, aliquots labeled MHT+ were submitted to an AMF (310 kHz, 305 Oe) for 25 min. Cell suspensions were then homogenized, and 10% trypan blue (*v*/*v*) was added to each aliquot to allow viable/non-viable cell counting in a Neubauer chamber.

### 2.9. Statistical Analyses

Unless otherwise stated, measurements were expressed as mean ± standard deviation (SD), and statistical analyses were performed by analysis of variance (ANOVA) followed by Tukey’s Post-Hoc test in GraphPad Prism 6. Statistically significant differences were assumed for *p* < 5%.

## 3. Results

### 3.1. Characterization of MnFe_2_O_4_ MNP

**Figure 1:**
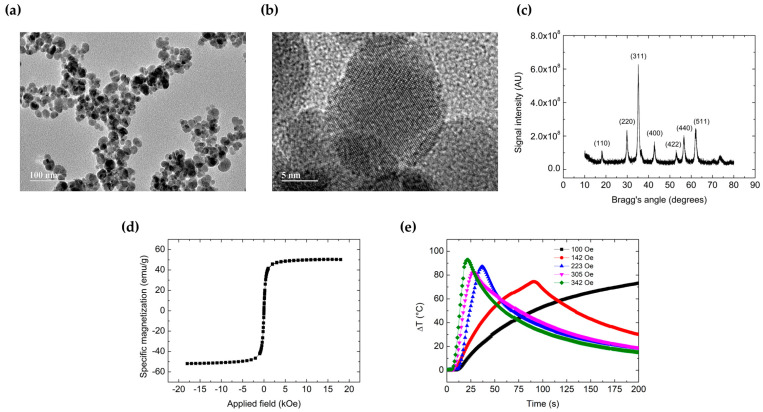
MnFe_2_O_4_ MNP characterization. (**a**,**b**) Representative transmission electron microscopy (TEM) and high-resolution (HR) TEM images, respectively, of the passivated manganese ferrite (MnFe_2_O_4_) magnetic nanoparticles (MNP). (**c**) X-ray diffraction (XRD) pattern and (**d**) specific magnetization profile of powder (dried) MNP samples. (**e**) Temperature profiles (relatively to room temperature, 25 °C) of MNP submitted to magnetic hyperthermia (MHT), with alternating magnetic field (AMF) frequency fixed at 310 kHz and varying amplitude (100–342 Oe).

Manganese ferrite (MnFe_2_O_4_) magnetic nanoparticles (MNP) comprise the core of our paclitaxel-loaded lipid-coated magnetic nanoparticles (PTX-LMNP). Therefore, they were fully characterized (Figure 1). Unless otherwise stated, they will be referred to simply as MNP in the following.

Figure 1a,b are representative transmission electron microscopy (TEM) images of the MNP. In terms of their shape, MNP synthesized via co-precipitation as in the described protocol are roughly spherical, measuring about 15 nm in diameter, as previously reported [50].

Figure 1c shows the X-ray diffraction (XRD) pattern of a typical powder (dried) sample of the original colloidal aqueous dispersion of MNP. The crystallite mean diameter, as calculated via Equation (1), was found to be 13.5 nm, in accordance with previous reports of our group for similarly synthesized MNP [50].

Figure 1d brings a representative magnetization profile of a powder sample of MNP, obtained via vibrating sample magnetometry (VSM). Specific saturation magnetization (about 50 emu/g) served as a reference for calculating the concentration (in terms of magnetic contents) of the corresponding MNP colloidal dispersions, as previously reported [50].

Figure 1e shows the behavior of a powder sample of MNP submitted to magnetic hyperthermia (MHT). Alternating magnetic field (AMF) amplitudes were varied within 100–342 Oe, with the field frequency fixed at 310 kHz. The initial slope (t→0) of the temperature profiles is proportional to the specific loss power (SLP, given in W/g) of the sample [27,50], a measure of its magnetothermal conversion efficiency under a given MHT setup. To be noted that higher temperatures could be attained in shorter timeframes (higher SLP) for higher field amplitudes. The exciting field was turned off as the temperature approached 100 °C to avoid damaging the temperature sensor.

For comparison purposes, Figure S1 brings Fe_3_O_4_ (magnetite) MNP characterization: XRD diffractogram, specific magnetization profile, as well as MHT temperature profiles.

### 3.2. Characterization of PTX-LMNP

**Figure 2:**
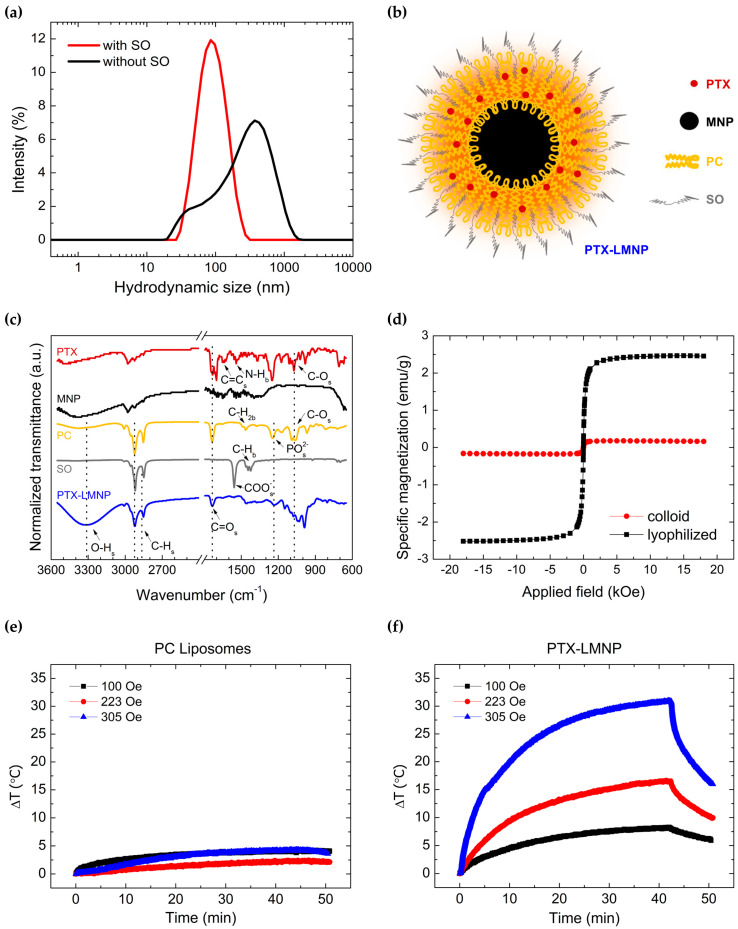
Characterization of PTX-LMNP. (**a**) Hydrodynamic size distributions obtained via DLS for samples with and without SO. (**b**) Schematic view of a paclitaxel-loaded lipid-coated magnetic nanoparticle (PTX-LMNP). PTX: paclitaxel; MNP: the core of manganese ferrite (MnFe_2_O_4_) magnetic nanoparticles (MNP); PC: the phosphatidylcholine coating; and SO: the sodium oleate surfactant molecules. (**c**) Normalized FTIR transmittance spectra for PTX-LMNP and its different components. (**d**) Specific magnetization profiles both for colloidally dispersed (fluid) and freeze-dried (lyophilized) PTX-LMNP samples. (**e**,**f**) Temperature profiles (relatively to room temperature, 25 °C) of PC liposomes (non-magnetic control sample) and PTX-LMNP, respectively, once submitted to magnetic hyperthermia (MHT), with alternating magnetic field (AMF) frequency fixed at 310 kHz and varying amplitude (100–305 Oe).

PTX-LMNP hydrodynamic size distributions for samples with and without SO are shown in Figure 2a. Their mean hydrodynamic diameter (D_H_) and polydispersity index (PdI) are summarized in Table 1. D_H_ was two-fold lower for samples with SO. Moreover, PTX-LMNP with SO were less polydispersed. Therefore, as depicted in Figure 2b, our optimal PTX-LMNP consisted of a core of magnetic MNP coated by a layer of phosphatidylcholine (PC), with SO as surfactant. Due to its lipophilic nature and relatively low solubility in water, PTX is expected to be found amidst the hydrophobic tails of the PC coating. Optimal mass proportions were, thus, set to 50:200:20:5 (MNP:PC:SO:PTX), as described in Section 2.4.

PTX entrapment efficiency (EE) and drug loading efficiency (DL) were calculated on the basis of HPLC results via Equations (3) and (4), respectively. The EE was found to be 81.2 ± 0.3%, whereas the DL amounted to 2.2 ± 0.6%.

No increase in D_H_ or PdI was observed for PTX-LMNP, neither for the colloidal dispersion stored at 4 °C for 30 days nor for the freeze-dried PTX-LMNP stored at 25 °C and reconstituted after 60 days (Table S1). In terms of the PTX cargo, a reduction of 8% was observed for the colloidal dispersion after 30 days, whereas no significant losses were observed for the freeze-dried PTX-LMNP reconstituted in water after 60 days (Figure S2).

Figure 2c shows the results of the Fourier Transform Infrared spectroscopy (FTIR) analyses carried out for PTX-LMNP and each of its components (PTX, MNP, PC, and SO) for wavenumbers at 650–3600 cm^−1^. To be noted that the transmittance spectra were normalized, and a break at 1800–2400 cm^−1^ was performed to highlight the most prominent absorption bands for each sample. Noteworthy, all prominent bands arising from the transmittance spectra of PTX, MNP, PC, and SO are also present in PTX-LMNP signal, which suggests that the nanocarrier was successfully assembled, as originally designed. Non-normalized FTIR spectra for PTX-LMNP and its components are provided in Figure S3.

The concentration of MNP in PTX-LMNP was obtained on the basis of specific magnetization profiles determine via vibrating sample magnetometry (VSM). Figure 2d shows the specific magnetization profiles of both aqueous-dispersed (fluid) and freeze-dried (lyophilized) PTX-LMNP samples. Similarly to the MNP magnetization profile (Figure 1d), the curves do not exhibit quasi-static hysteresis at room temperature, suggesting that the typical quasi-static superparamagnetic behavior of MNP [55] was preserved in PTX-LMNP. See Figure S4 for a zoomed view of Figure 2d around the origin for further details.

Figure 2e,f bring the temperature profiles of PC liposomes (non-magnetic control sample) and PTX-LMNP, respectively, when submitted to MHT. Temperatures were registered relatively to room temperature (25 °C). Devoid of magnetic constituents, PC liposomes are not excited by the AMF, and the observed temperature increase (less than 5 °C for all field amplitudes) is mostly due to heat release via eddy current losses (Figure 2e) [56]. In contrast, temperature variations within about 7.5 °C up to 30 °C were obtained as a result of dynamic (AC) hysteresis losses [57], due to the interaction between the AMF and PTX-LMNP magnetic core (Figure 2f).

### 3.3. PTX-LMNP Distribution in Porcine Ear Skin

**Figure 3:**
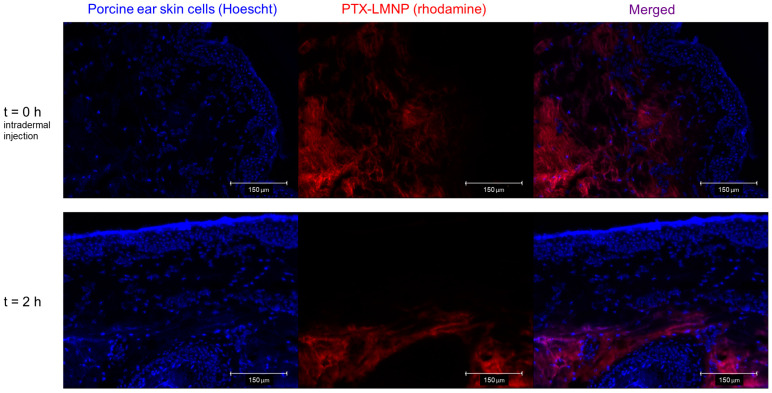
PTX-LMNP biodistribution in porcine ear skin. Representative fluorescence images of Hoechst-stained (blue fluorescence) porcine ear skin cells immediately after intradermal administration of rhodamine-labeled (red fluorescence) PTX-LMNP (0 h) and two hours later (2 h). Initially, at the interface between the epidermis and the dermis, PTX-LMNP diffuses to deeper layers of the dermis.

Fluorescence images of PTX-LMNP biodistribution in porcine ear skin are shown in Figure 3. Hoechst-stained epithelial cells appear in blue, whereas rhodamine-labeled PTX-LMNP appear in red. Right after intradermal injection, some PTX-LMNP could be found within the interface between the epidermis and the dermis (Figure 3, 0 h). In contrast, 2 h after injection, PTX-LMNP were no longer found in the epidermis or in its interface with the dermis, but only in deeper layers of the dermis (Figure 3, 2 h).

### 3.4. PTX-LMNP In Vitro Drug Release Profile

**Figure 4:**
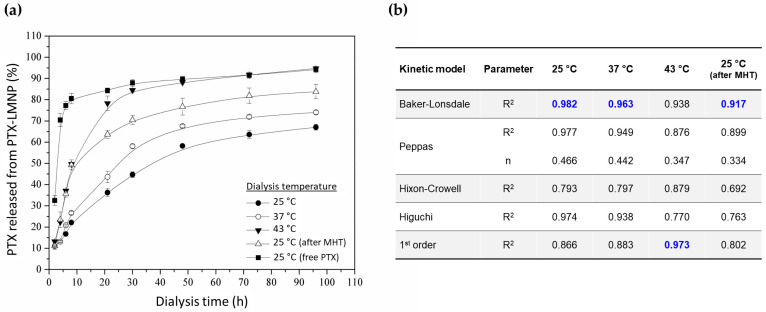
PTX release kinetics from PTX-LMNP. (**a**) PTX release profiles for different temperatures (25, 37, and 43 °C), as well as for a sample first submitted to MHT, and then left for dialysis at room temperature (25 °C). Free PTX was adopted as a control sample. (**b**) Coefficients of determination (R^2^) for different models of drug release kinetics after fitting the corresponding model equations to the experimental data. The parameter n represents the exponent of release (related to the release mechanism) of Peppas’ model [58]. R^2^ coefficients closest to unit are highlighted in blue.

Figure 4a shows the in vitro cumulative PTX release profiles for PTX-LMNP samples submitted to dialysis at 25, 37, and 43 °C for 96 h (4 days), as well as for PTX-LMNP samples that first underwent MHT, and then were submitted to dialysis at 25 °C for 96 h for PTX release assessment at scheduled times. Free PTX was used as a control sample at 1 mg/mL—the same concentration carried by a typical PTX-LMNP.

About 70% of the free PTX control sample reaches the receptor chamber within the first 4 h of experiment, and about 95% within 96 h. PTX percentual release from PTX-LMNP after 8 h of experiment could be doubled, relatively to the sample kept at 25 °C, either by (1) increasing the incubation temperature up to 43 °C or (2) applying MHT (310 kHz, 305 Oe) prior to the drug release assay.

Figure 4b summarizes the different attempts to fit the cumulative drug release data to five distinct models of drug release kinetics. Considering the coefficients of determination associated to the different fittings, Baker and Lonsdale’s model seems to be the best option to predict PTX release from PTX-LMNP over time for all five experimental setups, although first-order kinetics better models PTX release from PTX-LMNP at 43 °C.

### 3.5. PTX-LMNP Cytotoxicity against B16F10 Melanoma Cells

**Figure 5:**
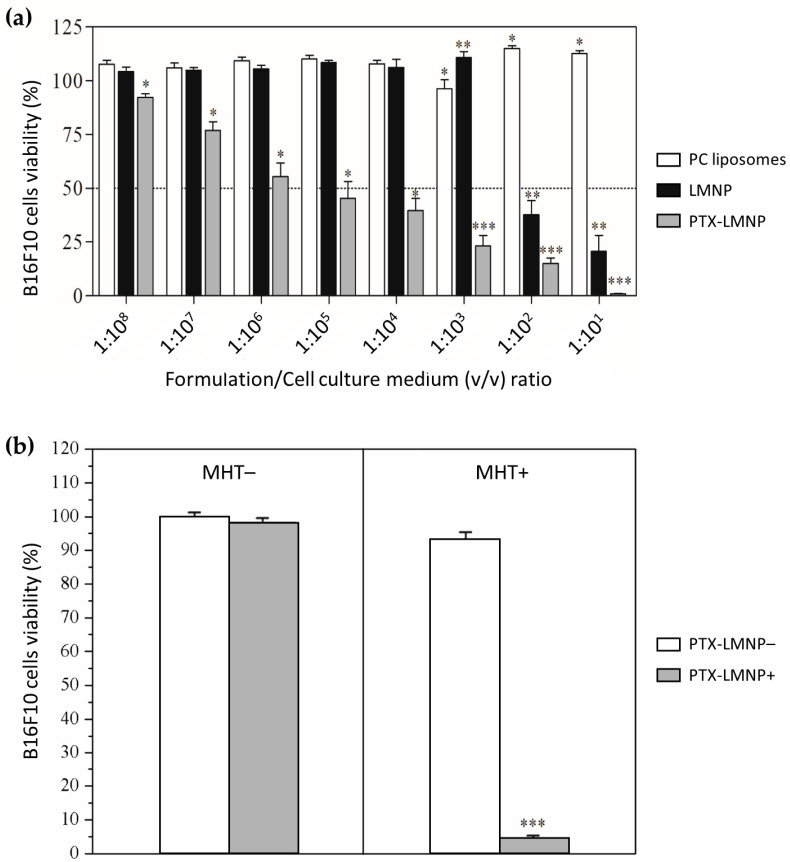
PTX-LMNP against B16F10 melanoma cells. (**a**) Cell viability 48 h after incubation with PC liposomes, LMNP, or PTX-LMNP samples. (**b**) Cell viability 1 h after incubation with or without PTX-LMNP (PTX-LMNP+ or PTX-LMNP− samples, respectively), followed or not by MHT (MHT+ or MHT− samples, respectively). * *p* < 0.05, ** *p* < 0.01, *** *p* < 0.001.

Figure 5a shows the results of the cytotoxicity assay (neutral red uptake, formulations incubated for 48 h with B16F10 cells). No significant decrease on cell viability was observed for PC liposomes (control samples, without PTX). Similarly, LMNP were cytotoxic only at high concentrations (IC_50_ ≈ 1:230 *v*/*v*, corresponding to about 14,000 ng/mL of MNP). In contrast, significant cell death was observed for PTX-LMNP, even at low concentrations (IC_50_ ≈ 1:10^6^ *v*/*v*, equivalent to approximately 0.4 ng/mL of PTX or 3.2 ng/mL of MNP).

Figure 5b summarizes the results of the experiment designed to verify the influence of MHT on PTX-LMNP cytotoxicity against B16F10 cells. Cell viability decreased to less than 5% for aliquot MHT+ PTX-LMNP+, whereas no significant differences in cell viability were observed for the other aliquots within the short timeframe of the experiment, i.e., 1 h of incubation followed or not by 25 min of MHT (Figure 5b).

## 4. Discussion

In previous works, we have already explored the controlled release of model drugs from nanostructures eventually triggered by magnetic hyperthermia (MHT). For instance, rapamycin was successfully encapsulated with Fe_3_O_4_ (magnetite) magnetic nanoparticles (MNP) within poly(lactic-co-glycolic acid) (PLGA) polymeric nanocapsules and nanospheres [59]. Additionally, paclitaxel (PTX) was incorporated to solid lipid nanoparticles composed by glyceryl monostearate (GMS) and phosphatidylcholine (PC) with magnetite cores and further stabilized by Pluronic F-68 [15]. Herein, we report the development of PTX-loaded lipid-coated magnetic nanoparticles (PTX-LMNP) to explore the potential of MHT-triggered release on treating skin cancer (B16F10 melanoma) by dual chemo-MHT therapy.

The delivery strategy herein reported was designed as a bioinspired synthetic analog of the naturally occurring magnetosomes [43,44,45]. This strategy has also captured the attention of other authors. For instance, magnetite MNP were embedded within a glyceryl trimyristate solid matrix as a platform for treating colon cancer via MHT [60]. Magnetite MNP embedding near infrared (NIR) fluorophores have also been coated with galactosyl and targeting moieties to enable dual-modal (fluorescence/magnetic resonance) imaging of hepatocellular carcinomas [61]. Moreover, 1,2-dipalmitoyl-sn-glycero-3-phosphocholine (DPPC) and L-α-dipalmitoylphosphatidyl glycerol (DPPG) were used as the lipidic coating of magnetite MNP envisaging the MHT-induced thermo-responsiveness of the resulting delivery system [62]. Magnetite MNP were also coated with PC and cationic lipids, in association with transferrin, for MHT-controlled gene delivery [63]. Oleic acid has also been previously used as the lipidic coating of magnetite MNP for the MHT-triggered release of doxorubicin [64].

PTX-LMNP are schematically depicted in Figure 2b. The presence of Fe^+3^ ions on the surface of the passivated manganese ferrite (MnFe_2_O_4_) MNP enables the interaction with lipid molecules, such as PC. PC predominantly negative polar heads are attracted by the positive Fe ions on the surface of MNP, leading to the assembly of lipid layers around the magnetic cores. Sodium oleate (SO) grafting, with surfactant properties, stabilize the resultant particles, preventing their aggregation. Due to the lipophilic nature of PTX, it is entrapped within PC hydrophobic tails [65,66]. MnFe_2_O_4_ MNP was adopted as an alternative to the ubiquitous Fe_3_O_4_ MNP. Biocompatible and safe for medical use, MnFe_2_O_4_ MNP has been explored by our group as magnetite MNP surrogates, eventually outperforming magnetite in terms of MHT (Figure S1), and figuring as promising candidates for MRI as contrast agents [67,68].

Figure 2c displays the normalized FTIR spectra for PTX-LMNP and its components (PTX, MNP, PC, and SO). The non-normalized spectra can be found in Figure S3. The C-O stretching band around 1070 cm^−1^ is present in both PTX and PC spectra, as well as amidst a series of adjacent bands in the spectrum for PTX-LMNP in the 900–1200 cm^−1^ range. The phosphate (PO^−2^) stretching vibration band near 1230 cm^−1^, which is present in PC spectrum, can also be found in PTX-LMNP spectrum. Notably, the carboxylate (COO^-^) stretching band, identified in the SO spectrum near 1550 cm^−1^, correlates to an almost negligible peak in PTX-LMNP spectrum, suggesting that this functional group may be responsible for the attachment of SO to the PTX-LMNP structure. The carbonyl (C=O) stretching band, centered at 1730 cm^−1^, was identified for PTX, PC, as well as PTX-LMNP. Typical C-H stretching vibrations were also observed for PC, SO, and PTX-LMNP. A broad and relatively strong band in PTX-LMNP spectrum, starting at 3000 cm^−1^ and going up to 3600 cm^−1^, seemingly correlates to an equally broad (though relatively weaker) band within the PC spectrum, probably indicating that a relatively large amount of PC could be successfully attached to the final PTX-LMNP structure. Since the FTIR data were acquired at 650–4000 cm^−1^, the typical Fe-O stretching vibrations from MnFe_2_O_4_ octahedral and tetrahedral sites (usually 400–600 cm^−1^) could not be clearly identified, although they do stand out within the FTIR results of very similar samples produced by our group (e.g., Zn-doped MnFe_2_O_4_), as previously reported [69]. Taken together, these results indicate that the PTX-LMNP nanocarrier was successfully assembled, as designed.

The efficiency of MHT applications depends on several properties of magnetic nanoparticles, including their size, polydispersion, functionalization, spatial arrangement, and concentration [25,28]. Herein, we focused our investigation on the influence of concentration on the heating efficiency. In particular, the heat released by MnFe_2_O_4_ MNP aggregates under MHT seem to depend strongly on the collective magnetic relaxation [57].

The difference in specific saturation magnetization between aqueous-dispersed and freeze-dried PTX-LMNP (Figure 2d) accounts for the fact that the magnetization signal was divided by the whole sample mass—including the mass of water (dispersant) for the fluid sample. The concentration of magnetic material in PTX-LMNP samples can be determined on the basis of the specific saturation magnetization obtained for powdered MNP (50 emu/g, Figure 1d), as previously reported [50]. Freeze-dried PTX-LMNP specific saturation magnetization of 2.6 emu/g, thus, translates to approximately 10.4 mg/mL of MNP, while the 0.6 emu/g obtained for the aqueous-dispersed PTX-LMNP corresponds to about 2.4 mg/mL. Similar results were observed in our previous work [15].

Temperatures as high as 55 °C can be achieved by PTX-LMNP-mediated MHT within about 40 min (Figure 2f). Knowing that skin temperature is about 32 °C, only 4 min are needed to increase it to the hyperthermia range of 43–46 °C, consequently promoting the desired therapeutic effects [36]. Note that the thermal dose can be tuned by controlling both the magnetic field amplitude and frequency, as well as the time of treatment, enabling local MHT-triggered release, while avoiding patient harm in a clinical scenario [36].

PTX-LMNP-facilitated diffusion through the skin (Figure 3) can be explained by their lipid composition. PTX-LMNP structural phospholipids and surfactants ease their diffusion through the different skin layers by enabling interactions with local tissue lipids and proteins [70]. Indeed, once delivered intradermally, we observed that PTX-LMNP migrate from the epidermis-dermis interface to deeper layers of the dermis. Therefore, PTX-LMNP are potentially able to deliver their therapeutic benefits to skin cancers established either within the epidermis, deeply in the dermis, or within both skin layers [71].

PTX-LMNP specific loss power (SLP) was found to be 37.8 W/g (with field frequency and amplitude set to 310 kHz and 305 Oe, respectively, and sample volume of 1 mL at 3.5 mg/mL, Figure 2f). Though higher SLP values can be found in the literature [72], PTX-LMNP SLP was high enough to efficiently trigger PTX release (Figure 4). Indeed, the temperature increase changes the molecular dynamics of the phospholipids in PTX-LMNP, increasing the lipid layer fluidity and permeability [73], thus favoring release of the drug cargo [74]. After fitting the experimental data to five different models, Baker–Lonsdale’s model for drug release from spherical matrices reasonably accounted for the release kinetics of all the different experimental setups. Noticeably, MHT-triggered release at 25 °C was comparable to PTX release kinetics at 43 °C, especially within the first 10 h (Figure 4). Note, however, that the release profiles in Figure 4 account both for restricted diffusion, within the dialysis bag, and free diffusion, outside the dialysis bag, where aliquots were then sampled at scheduled times for PTX quantitation via HPLC.

Baker–Lonsdale’s model has been most commonly applied to describe drug release from microcapsules and microspheres [58], but it also accounts for slow sustained release from spherical matrices [75]—which is the case depicted in Figure 4a for PTX-LMNP. Hydrophobic PTX molecules tend to be slowly released from the lipidic PC layer coating PTX-LMNP, to which they are attracted through hydrophobic interactions. Solubility and diffusion within the matrix, prior to release, are taken into account by Baker–Lonsdale’s model, but not by the first-order model, whose sole assumption is that release rates are proportional to the remaining concentration of the drug in the matrix. Temperature-induced phenomena might corroborate this assumption, since R^2^ more closely approached 1 for a first-order fit only when the experiment was carried out at 43 °C (Figure 4b).

Results of the cytotoxicity (neutral red uptake) assay suggest that B16F10 cell death is expected to be predominantly due to the chemotherapeutic action of PTX (Figure 5a). Additionally, although MNP were shown to be non-cytotoxic at concentrations as high as 0.1 mg/mL [76], some toxicity might occur at higher concentrations, as per the observed LMNP cytotoxicity profile. Noteworthy, the half-maximal inhibitory concentration (IC_50_) value obtained for PTX-LMNP (0.4 ng/mL) was nearly 340 times lower than that reported for the commercial formulation Taxol^®^ (137 ng/mL) [77] and remarkedly 142,500 times lower than that of free PTX (57 µg/mL) [78].

Significant cytotoxicity against B16F10 cells was observed for cells treated with both PTX-LMNP and MHT (PTX-LMNP+ MHT+ aliquot, Figure 5b). In contrast, no significant cytotoxicity was observed for cells treated only with PTX-LMNP (PTX-LMNP+ MHT−).

One drawback of this experiment, however, is that the effect of MHT in isolation cannot be determined. An additional control sample would be needed, either MNP or LMNP, at the same concentration of PTX-LMNP, in terms of magnetic contents, submitted to MHT in presence of B16F10 cells. We would then be able to determine whether B16F10 cells are dying exclusively due to the chemotherapeutic effect of PTX, or if cell death is a result of the combined effect of MHT and PTX, eventually in synergy. Nevertheless, in a previous work, we showed that B16F10 cells were resistant to hyperthermia temperatures up to 4 h of incubation [50]. This suggests that, in the current study, cells would be dying because of PTX.

Nevertheless, according to Figure 4a, PTX release from PTX-LMNP is too slow, even under the influence of MHT. Indeed, about 50% of the PTX cargo is released only after approximately 10 h after the beginning of the experiment. However, one might notice that both restricted diffusion (inside the dialysis bag) and free diffusion (outside the dialysis bag) are taken into account in Figure 4a. By contrast, in Figure 5b, there is no dialysis bag in the experimental setup, meaning that the whole PTX cargo released, whether or not under the influence of MHT, is immediately allowed to interact with the B16F10 cells, eventually exerting its cytotoxic action.

Still, according to Figure 5a, a 10% *v*/*v* concentration, as employed for the experiment in Figure 5b, seems to be intrinsically cytotoxic. However, the timeframes of both setups must be considered: in Figure 5a cytotoxicity is evaluated for 48 h (long-term assay), in contrast with Figure 5b, for which cytotoxicity is evaluated for no longer than 1 h 30 min (short-term assay). In this short-term assay, Figure 5b clearly shows that PTX-LMNP are not intrinsically cytotoxic, while significant cytotoxicity is observed under MHT.

Additionally, the volume adopted for the experiment of Figure 5b (10 mL) is significantly higher than that of a well in a 96-well plate, where PTX-LMNP are allowed to interact with B16F10 cells for the experiment of Figure 5a. Increased interaction with the formulation, allied to an increased incubation period, might also have contributed to the differences observed between the two experiments.

Moreover, the 10 mL might obscure the effect of temperature during the experiment, since the temperature variation of the ensemble at the macroscopic scale for these conditions is negligible. However, at the microscopic and at the nanoscopic scales, a localized MHT-mediated increase in temperature might have altered the PC-coating fluidity, favoring PTX release within the short timeframe of the experiment in Figure 5b, thus favoring a cytotoxic action. Indeed, 95% of cell death was observed within 1 h of incubation with PTX-LMNP followed by 25 min of MHT, while no significant reduction in cell viability was observed for cells incubated with PTX-LMNP but not submitted to MHT. Similarly, other authors also report a significant improvement in chemotherapy outcomes as a result of its combination with MHT [79,80,81].

Taken together, our results indicate that PTX-LMNP-mediated combined chemo-MHT therapy stands out as a promising strategy against melanoma. Prospectively, we envisage the elucidation of the mechanisms underlying the cytotoxicity induced by the combination of chemotherapy and MHT [82,83,84,85]. In vivo experiments will certainly challenge the in vitro results herein reported, although other authors have already corroborated the benefits of chemo-MHT and radiation-MHT combined therapy in pre-clinical assays using analogous drug-heat delivery systems [50,70,86,87,88].

## 5. Conclusions

Paclitaxel-loaded magnetic-lipid nanoparticles (PTX-LMNP) were developed envisaging the combined chemo-magnetic hyperthermia therapy of melanoma. With hydrodynamic diameters around 90 nm, PTX-LMNP consist of manganese ferrite (MnFe_2_O_4_) magnetic nanoparticles (MNP) coated by phosphatidylcholine (PC). With surfactant properties, sodium oleate (SO) successfully endowed PTX-LMNP dispersions with colloidal stability. Due to the hydrophobic nature of PTX, its entrapment efficiency (EE) within PC hydrophobic tails was greater than 80%. PTX-LMNP rapidly diffuse across different skin layers, due to interactions between its lipidic cloak with the epithelial tissue lipids and proteins. In vitro drug release profiles revealed that temperature modulates PTX release, with higher temperatures correlating with higher release rates. Magnetic hyperthermia (MHT) triggers and consequently enhances PTX cumulative release. PTX-LMNP cytotoxicity against B16F10 melanoma cells is concentration-dependent, with an IC_50_ corresponding to 0.4 ng/mL of PTX, significantly lower than the IC_50_ reported for free PTX and for PTX-loaded nanostructured formulations. Cytotoxicity was strikingly higher when PTX-LMNP were submitted to MHT, which suggests that the dual chemo-MHT therapy mediated by intratumorally injected PTX-LMNP stands out as a promising alternative to efficiently deliver PTX to melanoma cells, consequently reducing systemic side effects commonly associated with conventional chemotherapies.

## Figures and Tables

**Table 1:** PTX-LMNP size and polydispersity × SO contents.

Sample	D_H_ (nm)	PdI
without SO	186 ± 1	0.50 ± 0.15
with SO	90 ± 1	0.26 ± 0.03

D_H_ = mean hydrodynamic diameter, PdI = polydispersity index, SO = sodium oleate.

## Data Availability

Not applicable.

## Supplementary Materials

The following supporting information can be downloaded at: https://www.mdpi.com/article/10.3390/pharmaceutics15030818/s1, Figure S1: Fe_3_O_4_ MNP characterization; Figure S2: PTX-LMNP stability assay; Figure S3: Non-normalized FTIR spectra; Figure S4: PTX-LMNP magnetization within −100 to 100 Oe; Table S1: PTX-LMNP stability assay data.
