# Supplementary material for: Paclitaxel-Loaded Lipid-Coated Magnetic Nanoparticles for Dual Chemo-Magnetic Hyperthermia Therapy of Melanoma
Source: Pharmaceutics. 2023 Mar 2;15(3):818. doi: 10.3390/pharmaceutics15030818 (PMC10055620; doi:10.3390/pharmaceutics15030818)
Supplement: Supplementary file 1 [file pharmaceutics-15-00818-s001.zip › pharmaceutics-2164918-supplementary.pdf]

Supplementary Materials

Paclitaxel-Loaded Lipid-Coated Magnetic Nanoparticles for Dual Chemo-Magnetic Hyperthermia Therapy of Melanoma

Relton R. Oliveira [1], Emílio R. Cintra [1], Ailton A. Sousa-Junior [1], Larissa C. Moreira [2], Artur C. G. da Silva [2], Ana Luiza R. de Souza [1], Marize C. Valadares [2], Marcus S. Carrião [3], Andris F. Bakuzis [3,4] and Eliana M. Lima [1,4,*]

[1] FarmaTec—Laboratory of Pharmaceutical Technology, School of Pharmacy, Federal University of Goiás, Alameda Flamboyant, Qd. K, Ed. LIFE, Parque Tecnológico Samambaia, Goiânia 74690-631, Brazil

[2] ToxIn—Laboratory of Education and Research in In Vitro Toxicology, Federal University of Goiás, Alameda Flamboyant, Qd. K, Ed. LIFE, Parque Tecnológico Samambaia, Goiânia 74690-631, Brazil

[3] Physics Institute, Federal University of Goiás, Avenida Esperança, s/n, Campus Samambaia, Goiânia 74690-900, Brazil

[4] CNanoMed—Nanomedicine Integrated Research Center, Federal University of Goiás, Alameda Flamboyant, Qd. K, Ed. LIFE, Parque Tecnológico Samambaia, Goiânia 74690-631, Brazil

* Correspondence: emlima@ufg.br

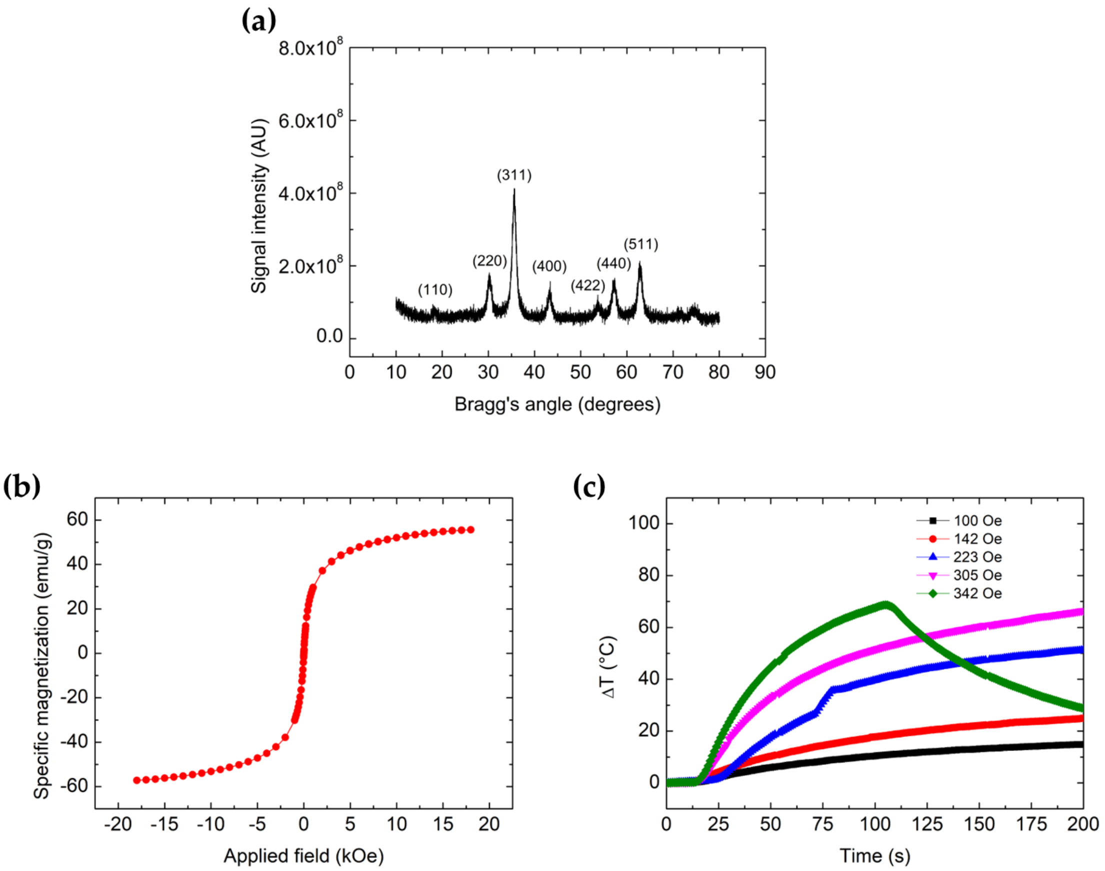

Figure S1. Fe_3O_4 MNP characterization. (a) X-ray diffraction (XRD) pattern and **(b)** specific magnetization profile of powder (dried) MNP samples. **(c)** Temperature profiles (relatively to room temperature, 25 °C) of MNP submitted to magnetic hyperthermia (MHT), with alternating magnetic field (AMF) frequency fixed at 310 kHz and varying amplitude (100-342 Oe).

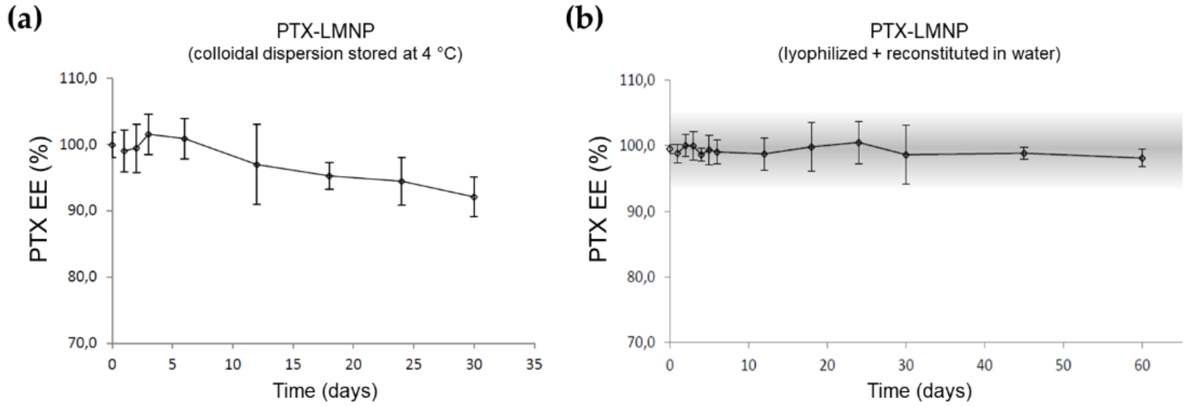

Figure S2. PTX-LMNP stability assay. Paclitaxel (PTX) entrapment efficiency (EE) of PTX-loaded lipid-coated magnetic nanoparticles (PTX-LMNP) over time for: **(a)** Colloidally dispersed PTX-LMNP stored at 4 °C up to 30 days; **(b)** Lyophilized PTX-LMNP stored at room temperature (25 °C) and reconstituted in water after up to 60 days.

Table S1. PTX-LMNP stability assay data.

**Sample**	**Elapsed time (days)**	**D_H (nm)**	**PdI**	**PTX EE (%)**
PTX-LMNP (fluid)	0	90.1	0.24	84.5 ± 1.6
	30	90.6	0.24	78.0 ± 2.4
PTX-LMNP (lyophilized)	0	90.0	0.26	80.6 ± 0.6
	60	90.6	0.25	79.5 ± 1.1

D_H = mean hydrodynamic diameter, **PdI** = polydispersity index, **PTX** = paclitaxel, **EE** = entrapment efficiency.

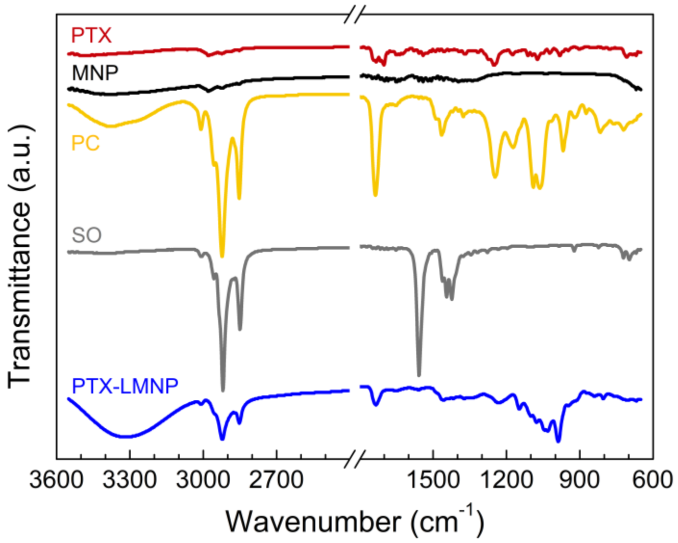

Figure S3. Non-normalized FTIR spectra. Transmittance spectra for PTX-LMNP and its different components.

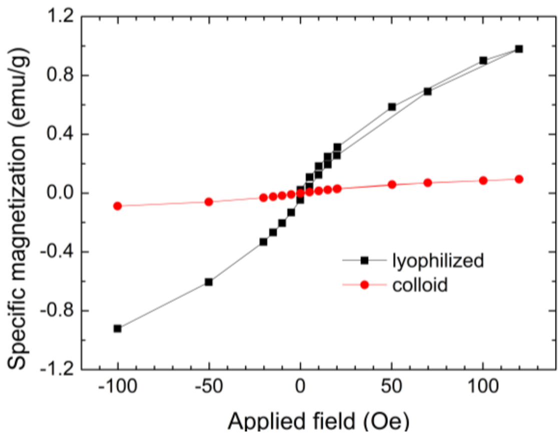

Figure S4. PTX-LMNP magnetization within -100 to 100 Oe. Zoom around the origin for the magnetization curves shown on **Figure 2c** of the main text, highlighting the negligible hysteresis (superparamagnetic behavior) under quasi-static Vibrating Sample Magnetometry (VSM) both for freeze-dried (lyophilized) and colloidal PTX-LMNP samples. Under AC excitation, however, a dynamic hysteresis loop is formed, which explains the heating observed when these samples are submitted to Magnetic Hyperthermia (MHT). To be noted that the field units are in Oe (as opposed to kOe, in the main text).
